# A study of the mediating effect of social support on self-disclosure and demoralization in Chinese older adult homebound breast cancer patients

**DOI:** 10.3389/fpsyg.2024.1365246

**Published:** 2024-04-17

**Authors:** Meifeng Liu, Fawei Qin, Deyu Wang

**Affiliations:** ^1^Department of Gastrointestinal Surgery, Shandong Provincial Hospital Affiliated to Shandong First Medical University, Jinan, China; ^2^Department of Oncology Minimally Invasive Surgery, Shandong Provincial Hospital Affiliated to Shandong First Medical University, Jinan, China

**Keywords:** older adults, homebound, breast cancer, self-disclosure, social support, demoralization, mediating effect

## Abstract

**Purpose:**

Demoralization is common in older adult homebound breast cancer patients, seriously affecting their quality of life. This study aimed to investigate the demoralization of older adult homebound breast cancer patients and to analyse the mediating effects of social support between self-disclosure and demoralization.

**Methods:**

The study enrolled 368 older adult homebound breast cancer patients reviewed in outpatient clinics of three hospitals from January 2022 to August 2023. A questionnaire survey was conducted using the general information questionnaire, the distress disclosure index (DDI), the social support revalued scale (SSRS), and the demoralization scale (DS). Path analysis was conducted to test the hypothesised serial mediation model.

**Results:**

The total scores of self-disclosure, social support, and demoralization were 37 (25–42), 34 (19–48.75), and 46.5 (35–68), respectively. The results indicated a positive correlation between self-disclosure and social support (*p* < 0.01). In contrast, a statistically significant negative correlation was observed between self-disclosure, social support, and various demoralization dimensions (*p* < 0.01). Social support played a partial mediation effects between self-disclosure and demoralization, indirect effect =0.6362, SE = −0.591, 95% CI (−0.785 ~ −0.415); Self-disclosure direct effect demoralization, direct effect =0.3638, SE = −0.337, 95% CI (−0.525 ~ −0.144); total effect, SE = −0.929, 95% CI (−0.945 ~ −0.904).

**Discussion:**

Social support a partial mediated between self-disclosure and demoralization in Chinese older adult homebound breast cancer patients. Clinical staff should focus on developing a social support system for Chinese older adult homebound breast cancer patients, encouraging patients to reveal their minds, and providing psychological counselling to enhance self-confidence and rebirth from adversity.

## Background

Breast cancer has become the most common cancer type worldwide (Sung et al., 2021). According to the latest Cancer Statistics Report of 2021, it seriously threatens the lives and health of the global female population (Siegel et al., 2021). Worldwide statistics highlight that approximately 40% of breast cancer cases occur in patients aged 65 years and above, with expectations that this will increase as the population gets older (Aladwani et al., 2023). Breast cancer is treated with surgical excision, radiation therapy, and chemotherapy. Rehabilitation of breast cancer is not a short-term process, but it requires long-term persistence, and it will cause different degrees of disturbance to the life and work of patients in the treatment and rehabilitation process. Approximately 50% of breast cancer patients suffer from psycho-psychological problems (Wells et al., 2022). In China, older adults who have entered the recovery phase of an illness are subjected to complete the subsequent rehabilitation process at home. The process of diagnosis, treatment, and rehabilitation of older adult breast cancer patients relies mainly on supportive family care and financial support (Tang et al., 2024). Most older adults have fewer financial resources and cannot fully afford all medical expenses; their physical endurance has significantly decreased due to their increasing age and suffering from cancer. As a result, they cannot complete the cancer treatment process independently. All of the above reasons may lead to poor treatment adherence in Chinese older adult breast cancer patients compared with young and middle-aged breast cancer patients (Zhou et al., 2024). This can lead to a poor physical prognosis for older adults and can also have a serious impact on the psychological and mental health of older adults (Lin et al., 2023).

Emotion regulation has been defined as “the processes by which individuals influence which emotions they have, when they have them, and how they experience and express these emotions” (Gross, 2015). Emotion regulation, the experience, processing, and modulation of emotional responses are necessary to manage the emotional stressors common in cancer patients (de Ridder et al., 2008). Optimizing emotion regulation promotes adaptation in the presence of aversive stressors (Gratz, 2004). The inability to effectively manage emotions triggered by a health event can diminish self-care activities and impact mental and physical health (Appleton et al., 2013). Demoralization is a syndrome in which the patient feels helpless and hopeless, has impaired self-esteem, and has a loss of purpose in life when facing chronic stress (Ting-Gang Chang et al., 2022), seriously affecting their quality of life and even leading to the idea of lightening their life (Tang et al., 2015; Zheng et al., 2022). Emotion regulation theory (Cisler et al., 2010) suggests that emotion can be regulated during self-representation through a series of strategies. Self-disclosure character, willingness, and ambivalence may all influence the psychological adjustment level of the individual (Li et al., 2017a; Perndorfer et al., 2019). Self-disclosure—an important way of maintaining mental health and an important avenue of psychotherapy—enables individuals to regain confidence and self-esteem through the disclosure of thoughts and feelings about traumatic events (Tsumura et al., 2023). A qualitative study of breast cancer peer support suggested that the ability to appropriately share a peer’s own experience of breast cancer was perceived as an important component of effective helping (Truong et al., 2019). With the arrival of the aging population, one of the problems resulting from the increase in the number of single-person households and older couple households is the decrease in opportunities for conversation within the household. A survey shown that: only about 65% of older people living alone engage in conversation every day, which was clearly lower than the more than 90% of older couple households and households with children living together (Igarashi et al., 2022). The survey of Truong et al. shown that peer self-disclosure can provide relief for older adults with depression. They suggested training and supervision in appropriate self-disclosure should be provided to peers to ensure purposive use (Truong et al., 2019).

The good venting of bad emotions requires spiritual support from family, units, and society. The supportive care theory (Philip et al., 2018; Krishnasamy et al., 2023) states that the supportive care needs of cancer patients may include physical, informational, emotional, social interaction, and psychological aspects. It emphasizes that patients should be provided with continuity of care appropriate to their needs. Social support, as an external resource, improves psychological resilience in cancer patients and serves as a protective factor for demoralization syndrome (Vehling et al., 2013), and the mechanism of its influence may be linked to supportive care theory. The proportion of the world’s population over 60 will nearly double from 12 to 22% between 2015 and 2050 (Bai et al., 2020). In the contemporary times when population aging becomes a pressing global concern, social support is more meaningful for older adults. Based on assessing a large amount of research, it is shown that supportive relationships protect us from a multitude of mental health problems, but also that the absence of supportive relationships increases the risk of dying from various diseases (Bai et al., 2020; Firouzbakht et al., 2020). The concern of family members and the comfort and support of friends and coworkers can help enhance the social belonging sense of the patient (Ji et al., 2019; Mo et al., 2022). Wu and Lu (2017) 19 used data from the 2011–2012 China Health and Retirement Longitudinal Study (CHARLS) to discuss the impact of children’s informal care on the health behaviors of elderly people with chronic diseases through the propensity score matching method and found that children’s informal care can improve the health behaviors of the elderly and thus improve elderly health. Tang et al. surveyed 449 participants over the age of 60. Multiple regression was used to explore moderating effects of formal social support (FSS) and found that accompanied support, and number of intimate contacts had significantly positive effects on older health (Tang et al., 2022).

Self-disclosure can help individuals rebuild positive social sharing beliefs (Tajvar et al., 2018; Levi-Belz and Hamdan, 2023). Social support influences the self-disclosure of individuals (Bu et al., 2023; Ricciardi et al., 2023); adequate social support helps individuals maintain a favorable emotional experience and increase self-confidence. Although a certain correlation may exist between self-disclosure, social support, and demoralization, the relationship and mechanism of action of the three variables have not been reported before. This study combines emotion regulation theory with an in-depth study from the perspective of supportive care theory. We hypothesized that social support may mediate the relationship between self-disclosure and demoralization in Chinese older adult homebound breast cancer patients. This study investigates the relationship between self-disclosure, social support, and demoralization in older adult homebound breast cancer patients. The study hypothesized that social support might mediate between self-disclosure and demoralization in Chinese older adult homebound breast cancer patients and then inform the targeted nursing measure development to reduce demoralization in this group.

## Research design and methods

### Participants and procedures

The study subjects were 368 Chinese older adult homebound breast cancer patients (all study subjects were female) reviewed in the outpatient clinics of three hospitals: Shandong Provincial Hospital, Affiliated with Shandong First Medical University, Shenzhen People’s Hospital, and First People’s Hospital of Changzhou. A cross-sectional survey with simple sampling was conducted from January 2022 to August 2023 to increase the sample size and improve sample representativeness.

The inclusion criteria were as follows: (1) pathological diagnosis of breast cancer (stages 0, I, II, III, and IV); (2) at least 2 months of home rehabilitation; (3) age ≥ 60 years old; (4) subjects who obtained informed consent; (5) subjects with clear thinking, normal cognition, and unimpaired verbal expression; (6) awareness of their condition. The exclusion criteria were as follows: (1) combined with other malignant tumors or recurrence of breast cancer; (2) combined with myocardial infarction, heart failure, and other serious diseases; (3) other reasons affecting the implementation of the survey.

The researcher facilitated a pre-survey of 30 older adult homebound breast cancer patients in January 2022 and calculated the standard deviation of demoralization to be 17.26 points. This was a cross-sectional study, and the sample size required was estimated using the sample size formula for cross-sectional studies, *N* = (*u*_*1-*α/2_ σ/δ)^2^ (Hu et al., 2011), setting α = 0.05, *u*_*1-*α/2_ = 1.96, δ = 2, σ = 17.26,and *N* = 286. The minimum sample size was finally determined to be 343, combined with the fact that AMOS analysis generally requires more than 200 copies, and the expected dropout rate was set to 20%. 382 people participated in the questionnaire survey, and after questionnaire verification, 14 questionnaires were excluded, and the effective recovery rate of the questionnaires was 96.34%. A total of 368 patients were included in this study.

## Measures

### Sociodemographic and medical variables

Basic information, such as gender, age, marital status, educational level, monthly household income, and religious beliefs, was self-reported. Disease staging, pathological classification, and treatment plans were extracted from medical records.

### Distress disclosure index (DDI)

The DDI was used (Zhen et al., 2018), which consisted of 12 items and was scored by the Likert 5-level scoring method, with a total score of 12–60 points. The higher the DDI score, the higher the self-disclosure level. Typically, 12–29 points were low self-disclosure, 30–44 points were medium self-disclosure, and 45–60 points were high self-disclosure. The total Cronbach’s coefficient of the revised scale was 0.723 (Xu et al., 2024).

### Social support revalued scale (SSRS)

The SSRS (Zhang et al., 2023) contains a total of 10 items and consists of three dimensions: subjective support, objective support, and social support utilization. This scale has a full score of 66 points, with higher scores indicating a higher level of social support received, and social support is categorized into 3 levels: ≤22 points were low social support, 23–44 points were medium social support, 45–66 points were high social support (McNamara et al., 2021). The total Cronbach’s alpha coefficient was 0.920, which is widely used in China (Wang et al., 2024).

### Demoralization scale (DS)

DS was developed and compiled by Kissane et al. (2004) for cancer patients, and used to assess the status of demoralization in cancer patients in the past 2 weeks. It consists of 24 items in five dimensions: dysphoria (5 items), loss of meaning (5 items), helplessness (5 items), disheartenment (4 items), and sense of failure (5 items). Each item was rated on a 5-point Likert scale from 0 to 4, ranging from “Strongly Disagree” to “Strongly Agree,” and the total score ranged from 0 to 96. The cutoff was 30 points, and > 30 points was considered significant demoralization. The total Cronbach’s alpha coefficient was 0.740 (Battaglia et al., 2020).

### Data collection and analysis

This cross-sectional study was approved by the ethics committee of our hospital. Before the study, researchers were trained and assessed in a unified manner and were required to use unified guidelines in the survey process. This study was conducted using questionnaires in the form of stars. The researcher prepared the questionnaire through the Questionnaire Star program, formed a QR code for the Microsoft questionnaire, printed out a paper version, and posted it in the breast clinic to collect information. The questionnaire was completed during subsequent visits and was anonymous and voluntary. The first part of the questionnaire described the study’s purpose, and informed consent was obtained from all patients. The second part was the questionnaire body, which included DDI, SSRS, and DS. The survey was completed anonymously online, only once in Microsoft. The survey respondents completed the questionnaire independently. The research team members were responsible for reading and explaining the relevant items individually and guiding the patients to complete the questionnaire when encountering patients with reading and comprehension difficulties. The questionnaire was submitted after completion to ensure authenticity, collected, numbered, and double-checked. The exclusion criteria were as follows: questionnaires containing omissions and incompleteness, single answers, and contradicting content.

AMOS 23.0 and SPSS23.0 statistical software were used for data processing. Descriptive data are presented as mean ± SD for normally distributed variables and Md (P25, P75) for non-normally distributed variables. The enumeration data are expressed by the frequency and constituent ratios. In this study, social support, self-disclosure, and demoralization scores were non-normally distributed; therefore, the demographic characteristic scores were expressed by Md (P25, P75), conducting a correlation analysis using Spearman rank correlation analysis. In order to investigate whether SSRS has an impact as an independent predictor or a moderator on DS, a hierarchical multivariate regression analysis was performed. A value of *p* < 0.05 was considered statistically significant. Amos 23.0 was used to construct the structural equation model, the bootstrap was used to evaluate the direct and indirect effects, and the effects of each path were tested. All tests were two-sided, and a *p*-value of less than 0.05 was considered significant. Structural equation modeling was used to analyze the mediating mechanisms of social support, self-disclosure, and demoralization. Bootstrap was used to test the significance of the mediating effect; a mediating effect was considered significant if the 95% bias-corrected confidence interval for the indirect effect size did not contain zero. The fit of the hypothesized model to the observed data was determined based on the fit index. If the model fit was poor, the correlation between the error terms was added according to the modification indices (MI) (MacCallum et al., 1992), and the model was modified to improve the fit between the model and data.

## Results

### General information about patients

Table 1 showed that the age of the 368 participants was mainly 60–70 years old (73.10%). The education was elementary school and below (62.22%), and only 55 had a high school degree or above (14.95%). Among the religious beliefs, 84.78% had no religious beliefs, and 8.69% believed in Buddhism and Christian. Among the marital status, 73.10% were married. A total of 285 patients (77.44%) had medical insurance. The number of patients with stage II was 187 (50.81%). The monthly household income of 54.9% of patients was 5,000–10,000 RMB. Among 368 cases of breast cancer, 318 (96.6%) were invasive. Surgery were performed in 185 patients (50.27%), and chemotherapy were performed in 112 (30.43%).

**Table 1:** Demographic and clinical characteristics of participants.

Characteristics	Category	*N*	Percentage (%)
Age (years)			
	60 ~ 70	269	73.10
	﹥70	99	26.90
Education			
	Primary and below	229	62.22
	Junior high	84	22.82
	High school and above	55	14.95
Religion			
	No religion	312	84.78
	Christian buddhism	2	0.54
		30	8.15
	Others	24	6.52
Marital			
	Unmarried	5	1.36
	Divorce	94	25.54
	Married	269	73.10
Medical insurance			
	Yes	285	77.44
	No	82	22.28
Stage of breast cancer			
	Stage 0	21	5.71
	Stage I	27	7.34
	Stage II	187	50.81
	Stage III	112	30.43
	Stage IV	21	5.71
Monthly household income (RMB)			
	﹤5,000	106	28.8
	5,000 ~ 10,000	202	54.9
	﹥10,000	60	16.3
Pathological classification			
	Non invasive carcinoma	50	13.59
	Invasive carcinoma	318	86.41
Treatment plan			
	Surgery	185	50.27
	Chemotherapy	112	30.43
	Targeted therapy	71	4.62

### The scores and correlation analysis of self-disclosure, social support, and demoralization

The total DDI score was 37 (25–42), and the total SSRS score was 34 (19–48.75). The three dimensions were as follows: subjective support with 17 (12–28.75), objective support with 9 (5–12), and social support utilisation with 8 (4–10). The total DS score was 46.5 (35–68). The five dimensions were as follows: dysphoria with 9 (7–13), loss of meaning with 9 (7–14), helplessness with 9 (5–13), disheartenment with 10 (7–13), and sense of failure with 9 (6–13.75) (Table 2). Table 3 presented the correlation coefficients among self-disclosure, social support, and demoralization. The total scores of social support, self-disclosure, and demoralization of patients were significantly correlated (*p* < 0.01). Self-disclosure was positively correlated to social support (*r* = 0.870–0.889, *p* < 0.01), while self-disclosure and social support was negatively correlated to demoralization (*r* = −0.917–0.790, *p* < 0.01; Table 3).

**Table 2:** Scores of DDI, SSRS, DS.

Scale	Items	Actual range	M (P25-P75)
DDI	12	12–60	37 (25–42)
subjective support	4	8–32	17 (12–28.75)
objective support	3	1–22	9 (5–12)
social support utilization	3	2–12	8 (4–10)
SSRS	10	10–66	34 (19–48.75)
loss of meaning	5	0–20	9 (7–14)
dysphoria	5	0–20	9 (7–13)
disheartenment	5	0–20	10 (7–13)
helplessness	4	0–16	9 (5–13)
sense of failure	5	0–20	9 (6–13.75)
DS	24	0–96	46.5 (35–68)

**Table 3:** Bivariate correlations of study variable.

Items	1	2	3	4	5	6	7	8	9
1. Distress disclosure	1								
2. Subjective support	0.882^**^	1							
3. Objective support	0.889^**^	0.908^**^	1						
4. Social support utilization	0.870^**^	0.852^**^	0.902^**^	1					
5. Loss of meaning	−0.849^**^	−0.846^**^	−0.840^**^	−0.790^**^	1				
6. Dysphoria	−0.899^**^	−0.897^**^	−0.879^**^	−0.864^**^	0.881^**^	1			
7. Disheartenment	−0.917^**^	−0.900^**^	−0.882^**^	−0.841^**^	0.891^**^	0.929^**^	1		
8. Helplessness	−0.884^**^	−0.919^**^	−0.887^**^	−0.842^**^	0.869^**^	0.916^**^	0.912^**^	1	
9. Sense of failure	−0.908^**^	−0.914^**^	−0.892^**^	−0.850^**^	0.872^**^	0.935^**^	0.949^**^	0.946^**^	1

^**^p < 0.01.

### Structural equation model of self-disclosure, social support, and demoralization

The results of hierarchical multiple regression analysis are presented in Table 4. In the first step, age, education, medical insurance, monthly household income, marital, religion, stage of breast cancer, pathological classification and treatment plan were added into the model. In the second step, DDI were added as regressors. In the third step, SSRS were added as regressors. Model 1 accounted for 3.0% of the variance in DS, in which the main effects of education (*β* = 0.111, *p* < 0.05) and medical insurance (*β* = −0.112, *p* < 0.05) were significant in predicting the severity of DS. In model 2, the main effects of monthly household income (*β* = −0.052, *p* < 0.01), marital (*β* = −0.042, *p* < 0.05), religion (*β* = 0.057, *p* < 0.01), and DDI (*β* = −0.938, *p* < 0.001), were significant, which accounted for the additional 84.7% of variance in DS. In model 3, the main effects of monthly household income (*β* = −0.036, *p* < 0.01), DDI (*β* = −0.429, *p* < 0.001), SSRS (*β* = −0.558, *p* < 0.001) were significant, which accounted for the additional 5.3% of the variance in DS. Meanwhile, when DDI entered the model, the standardized regression coefficient of DDI increase from −0.938 to −0.429 (Table 4). The possibility that SSRS influences the relationship between DDI and DS may be the main reason for this result.

**Table 4:** Hierarchical multiple regression with DS as the dependent variable (*n* = 368).

Variables	Step 1		Step 2		Step 3	
	B	*β*	B	*β*	B	*β*
Age (years)	0.754	0.016	−0.160	−0.003	−0.287	−0.006
Education	3.204*	0.111*	1.070	0.037	0.684	0.024
Medical insurance	−5.708*	−0.112*	−1.354	−0.027	−0.599	−0.012
Monthly household income (RMB)	−0.441	−0.015	−1.519**	−0.052**	−1.047*	−0.036*
Marital	1.668	0.038	−1.860*	−0.042*	−0.842	−0.019
Religion	−1.859	−0.051	2.086**	0.057**	0.169	0.024
Stage of breast cancer	−1.314	−0.055	0.203	0.008	0.127	0.005
Pathological classification	−0.106	−0.002	0.736	0.012	−0.689	−0.011
treatment plan	−1.550	−0.056	−0.784	−0.028	−0.214	−0.008
DDI	/	/	−1.387***	−0.938***	−0.635***	−0.429***
SSRS	/	/	/	/	−0.683***	−0.558***
*F*	1.245		254.784***		430.294***	
*R*^2^	0.030		0.877		0.930	
Δ*R*^2^	0.030		0.847		0.053	

Variable assignment: Age(years), 60 ~ 70 = 1, ﹥ 70 = 2; Education, Primary and below = 1, junior high = 2, High school and above = 3; Medical insurance, yes = 1, no = 2; Monthly household income (RMB), ﹤ 5,000 = 1,5,000 ~ 10,000 = 2, ﹥ 10,000 = 3; Marital, unmarried = 1, divorce = 2, married = 3; Religion, No religion = 1, Christian = 2, Buddhism = 3, others = 4; Stage of breast cancer, Stage 0 = 1, Stage 1 = 2, Stage2 = 3, Stage 3 = 4, Stage 4 = 5; Pathological classification, non invasive carcinoma = 1, invasive carcinoma = 2; treatment plan, surgery = 1, chemotherapy = 2, targeted therapy = 3; DDI, SSRS were entered as the original value. Original value input; B: unstandardized regression weight, β: standardized regression weight; ΔR2:R2 change; ^*^p < 0.05; ^**^p < 0.01; ^***^p < 0.001.

**Figure 1:**
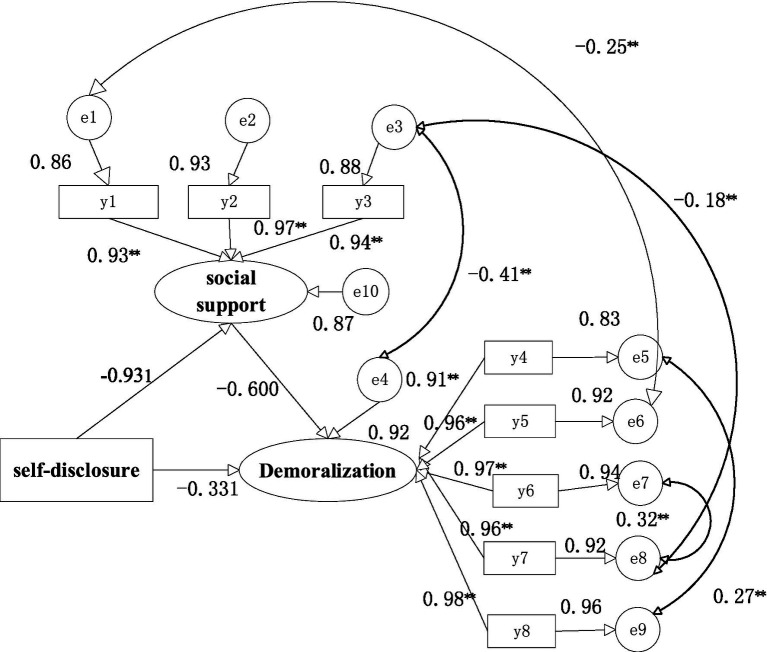
The map of the mediating effect. y1, subjective support; y2, objective support; y3, social support utilization; y4, dysphoria; y5, loss of meaning; y6, helplessness; y7, disheartenment;y8, sense of failure.^**^p < 0.01.

Through several literature and correlation analysis results, we hypothesised that self-disclosure and social support could, directly and indirectly, affect demoralization, respectively. The maximum likelihood method was used to fit the model structure, and the model was corrected according to the correction index. Model fit was assessed using various fit indices, including the chi-square (χ2) test of model fit, root mean square error of approximation (RMSEA), comparative fit index (CFI), goodness of fit (GFI), normed fit index (NFI) and Tucker-Lewis index (TLI). Indices were assessed using the following guidelines: 1 < χ2 /df < 3; RMSEA <0.08; GFI, CFI, NFI and TLI > 0.90 (Bu et al., 2023). The model fitting results were that χ^2^/df = 2.480, GFI = 0.972, RMSEA = 0.064, NFI = 0.992, CFI = 0.995, and TLI = 0.991, indicating that the fitting degree of the model was good (6) (Table 5). Figure 1 showed that social support can indirectly affect demoralization through self-disclosure. Table 6 summarised the path coefficients among the variables in detail. The mediating effect analysis showed that social support played a partial mediated the role in the self-disclosure and demoralization of older adult homebound breast cancer patients, with a mediating effect value, accounting for 63.62% of the total effect. Social support played a partial mediation effects between self-disclosure and demoralization, indirect effect =0.6362, SE = −0.591, 95% CI (−0.785 ~ −0.415); Self-disclosure direct effect demoralization, direct effect =0.3638, SE = −0.337, 95% CI (−0.525 ~ −0.144); total effect, SE = −0.929, 95% CI (−0.945 ~ −0.904).

**Table 5:** Model fitting index.

Model fitting index	CMIN/ DF	RMSEA	GFI	NFI	TLI	CFI
Standard or critical value	1 < NC < 3	﹤0.08	﹥0.9	﹥0.9	﹥0.9	﹥0.9
Results	2.480	0.064	0.972	0.992	0.991	0.995
Judgment of model fitness	Yes	Yes	Yes	Yes	Yes	Yes

**Table 6:** Mediating effect test results.

Path	*Estimate*	95% CI	*p* value	Effect (%)
Direct effect: self-disclosure → Demoralization	−0.337	−0.525 ~ −0.144	0.001^**^	36.38
Indirect effect: self-disclosure → Social Support → Demoralization	−0.591	−0.785 ~ −0.415	0.001^**^	63.62
Total effect	−0.929	−0.945 ~ −0.904	0.001^**^	–

^**^p < 0.01.

## Discussion

### The status of demoralization, self-disclosure and social support in Chinese older adult homebound breast cancer patients

Herein, the older adult homebound breast cancer patients had high demoralization scores, with a median score of 46.5, higher than the breast cancer patient scores studied by Hao et al. (2023) (43.29 ± 10.29) and Chang et al. (2022) (25.12 ± 14.89). This may be linked to the lower literacy level and age experience of older adult homebound breast cancer patients (Henry et al., 2023). Older adults are generally less educated than young and middle-aged adults. Individuals with higher education are generally more inclined to seek effective information resources and higher levels of disease-related health literacy when facing major diseases (Gao et al., 2023). However, she may have insufficient knowledge about disease prognosis and regression when an individual has a low education level and inadequate awareness to seek other resources and help actively (Liu et al., 2023). Consequently, they will be supported to a lesser extent, become more likely to lose the purpose and meaning of life, and experience a sense of meaninglessness, frustration, powerlessness, and increasing demoralization risk. The less-educated elderly population, who generally have lower incomes, cannot afford the high treatment cost, becoming more likely to experience higher demoralization levels. Conversely, their ability to take care of themselves has seriously declined with age, and usually, they are widowed, or their partners are suffering from illnesses; consequently, there is a relative lack of social support in caring for patients at home. These aspects substantially cause a serious psychological burden to patients, who feel that they are a burden to others and are very prone to emotional instability and a sense of failure, losing their confidence in treatment and life (Li et al., 2017b; Zhuang et al., 2022).

The median self--disclosure score of the older adult homebound breast cancer patients in this study was 37 (moderate level), lower than that of Zhu et al. (2023) (40.41 ± 7.34) for resident breast cancer patients. There are several reasons for the low self-disclosure scores of older adult homebound breast cancer patients. First, for age-related reasons, Zhu et al. (2023) found that the study population was 25–49 years old, which belonged to the middle-aged and young stage, whereas the study population was older adults aged over 60 years. The difference in age between the two groups may also be an important factor influencing the lower self-disclosure level of older adults with breast cancer in this study. Older adults are unwilling to accept that they need care because of their gradual loss of labor capacity, reduced self-care ability, and decreased economic income, but their self-esteem is still very strong, and they do not express their difficulties too much (Du et al., 2021). Second, due to cultural background-related reasons, Asian women are reserved and introverted, and when a female-related disease occurs, they are too shy to express it from their perspective, or they are worried that it will lead to discrimination by people around them; accordingly, they will suppress their misery. Third, due to disease response-related reasons, this may be correlated with mastectomy, radiotherapy, chemotherapy, and fear of cancer. Considering the damage to their image, patients will hide their inner thoughts of low self-esteem and worry, which is more likely to prevent venting of emotions and often result in a disorder of self-expression (Yang et al., 2022). Clinical staff should develop targeted measures in clinical treatment to increase levels of self-disclosure and help patients gradually return to daily life by seeking support from traumatic events.

In this study, the median social support score of older adult homebound breast cancer patients was 34 (moderate), lower than previously reported survey points (39.35 ± 9.51) (Jadidi and Ameri, 2022). This may be related to the fact that hospitalization and home care for breast cancer patients require more support and companionship from family members. In China, older adults mainly rely on the support and care of their children and spouses during illness. Under the current social environment of tight employment and great work pressure, Chinese older adults usually rely on their spouses to care for them (Liao et al., 2023); however, their spouses are usually not in good health. Consequently, the above reasons may contribute to the low social support for Chinese older adult breast cancer patients who are homebound. In clinical nursing, medical staff should strengthen the health education of older adult homebound breast cancer patients and their families, encourage family members to care more about the patients, and encourage patients to actively participate in social activities to strengthen their sense of social belonging.

In fact, under the new normal of COVID-19, China is committed to promoting the improvement of public health policies. Regular healthcare workers’ rural visit programs promote health consultation services for rural patients in the convalescence period and psychological disorder patients in the convalescence period. The development of mental health counseling services will largely alleviate the psychological pressure on older adult patients with homebound breast cancer. It is hoped that older adult homebound breast cancer patients can have healthy minds in the near future.

### Relationship between self-disclosure, social support, and demoralization

This study showed that social support of older adult homebound breast cancer patients was negatively correlated with demoralization, which was in general agreement with Kang et al. (2023), who concluded that social support of cancer patients was a risk factor for demoralization, and its change trend was the opposite to that of demoralization. Hong et al. (2022) reports social support (mean rs: −0.330) was negatively related to demoralization. Demoralization is a consequence of the interaction of physical, psychological, and social factors among cancer patients. They suggested factors such as social support with a significant effect should not be overlooked when designing an intervention to reduce demoralization. Chan et al. (2022) also provides an implication that those who have the least family support could be the most vulnerable group at risk of demoralization.

Thompson et al. (2013) conducted a two-year study on breast cancer patients and found that psychological problems increased as social support declined. Decreased social support can predict psychological problems in patients; the more emotional and informational support a patient has when facing various stresses, the less shock they experience. Medical social support acts as a good buffer and protects against demoralization. Social support of older adult homebound breast cancer patients was positively correlated with self-disclosure. This indicates that the more social support patients receive, the higher the self-disclosure level, which is consistent with Bu et al. (2023). Social support is crucial in sustaining treatment and promoting recovery (Li et al., 2017b). Emotional support provided by family and friends and information support provided by medical staff can help patients reveal their feelings and reduce their stress (Hancox et al., 2023; Orzechowska et al., 2023).

Our study displayed that self-disclosure in older adult homebound breast cancer patients was negatively correlated with demoralization, indicating that the lower the self-disclosure level, the lower the mood and the higher the demoralization level of the patient. When individuals, especially older adult homebound breast cancer patients, face unsolved difficulties, such as cancer horror, they may fear the character of “cancer.” They tend not to mention cancer when communicating with the outside world, cannot vent their negative emotions (Scandurra et al., 2022), and cannot adapt to new situations, increasing the demoralization level.

### The mediating effect of social support between self-disclosure and demoralization

Considering the analysis of hierarchical multiple regression, model 1 shown that the main effects of low education level and bad insurance were significant in predicting the severity of demoralization, which was consistent with the previous studies (Okado et al., 2014; Chang et al., 2022). The education level and medical insurance are usually related, people with low education level generally usually have not excellent medical insurance methods. Currently, there was no sufficient evidence showing that lower education led to more demoralization, and we speculated that it may be also related to self-disclosure. But more researches are needed. With lack of knowledge, lack of awareness of acquiring disease-related knowledge, fear of breast cancer, they were understandable that people may beyond expression, leading to more severe demoralization. Add DDI to model 2, Model 2 shown that the main effects of low monthly household income, unmarried or divorce, no religion, low DDI were significant in predicting the severity of demoralization. Some previous studies shown that low economic level and unmarried or divorce would affect the individual’s mental state, to some extent. She was very likely to lose the direction and guidance of life when he undergoes serious changes (Mohr and Huguelet, 2004; Nanni et al., 2018; Erdogan and Aydinoglu, 2023) when an individual has no religious belief. The effects of DDI was significant among those four. The results were in accord with the status quo of the corresponding demoralization, suggesting the important influence of self-disclosure on demoralization symptoms (Rusch et al., 2014). Research had shown self-disclosure with mental illness or anxious was associated with decreased negative effects of mental illness on quality of life (Corrigan and Rao, 2012). Add SSRS to model 3, Model 3 shown that the main effects of low monthly household income, low DDI, low SSRS were significant in predicting the severity of demoralization. The effects of DDI and SSRS were more significant among those three variable. It shown that the positive effect of social support will promote the release of mental stress, then alleviate symptom of demoralization, which was consistent with the previous studies (Vehling et al., 2013; Lo et al., 2023).

Our results indicated that social support completely mediated the relationship between self-disclosure and demoralization in older adult homebound breast cancer patients and that social support both directly and negatively predicted demoralization and indirectly and negatively influenced demoralization through self-disclosure. Older adult homebound breast cancer patients always worry that their lives and health are threatened, which adversely affects their quality of life and ability to plan their lives. Once there is a lack of listening, encouragement, and support from family, friends, and medical staff, the negative emotions of the individual will be unable to vent. In the long run, it can lead to anxiety and depressive conditions, demoralization (de Figueiredo et al., 2023), and even suicide (Fang et al., 2014). Conversely, with adequate social support, self-disclosure increases, and patients can express their emotions more positively to family, friends, and medical personnel. In this way, they can rationally utilize the resources around them to help them overcome difficulties, adapt quickly to their environment, and are less likely to experience demoralization. Consequently, healthcare professionals should provide a series of medical-social support for older adult homebound breast cancer patients to reduce demoralization and mobilize the supportive power of relatives and friends of the patients. Discharge continuation care can be conducted, such as follow-up visits to the homes of patients, applying for a breast cancer public number, and regularly uploading videos of postoperative functional exercises and precautions for radiotherapy and chemotherapy through the public number. This can help patients comfortably solve the difficulties of disease treatment and reduce the problem of psychological maladaptation.

### Study limitations

This study had some limitations. First, a cross-sectional design was applied to the present study, so these findings could not be used to construct formal causality or to identify the direction of causality between self-disclosure and demoralization. They need to be validated via longitudinal research to explore the trajectory of demoralization in patients at different times. Second, although this study collected data from three large general hospitals, simple sampling was used, requiring sample representativeness to be improved. We will try to improve the sample representativeness using random sampling methods and continue to increase the number of hospitals included in the study to expand the sample size. Third, psychological variables were mainly evaluated using self-report instruments, which might be subject to recall and response biases. Our study tried to minimize bias using self-disclosure, social support, and demoralization, which have been well-validated for application among subjects in China. Finally, our study focused only on the association between self-disclosure, social support, and demoralization. Further investigation is needed to explore other social psychology and emotional predictors for the level of demoralization in older adult homebound breast cancer patients, such as self-efficacy, psychological resilience, and family environment factors. Simultaneously, it is recommended to construct an intervention program for loss of ambition syndrome centered on self-expression and social support and conduct clinical empirical research to verify the mechanism of action among the three and improve the quality of clinical care.

## Conclusion

In summary, our findings suggest that the demoralization of Chinese older adult homebound breast cancer patients was generally at a higher level among the hospitals represented in the current study sample. Social support partially mediated the relationship between self-disclosure and demoralization in older adult homebound breast cancer patients, which was the first attempt to perform the relationship between psycho-social mediating resources and demoralization among Chinese older adult homebound breast cancer patients in our limited knowledge. This study found that social support can directly and negatively predict self-disclosure in older adult homebound breast cancer patients. Additionally, it indirectly and negatively affects demoralization through self-disclosure. Healthcare professionals should encourage patients to communicate with their family members and friends more often to increase their social support. Healthcare workers should help older adult homebound breast cancer patients express their bad moods and increase opportunities for self-disclosure actively while communicating and interacting with patients to provide them with disease-related professional information. The demoralization level of the patients was diminished by enhancing social support, raising the self-disclosure level. Ultimately, it promotes both physical and psychological recovery in older adult patients with homebound breast cancer.

## Data availability statement

The original contributions presented in the study are included in the article/supplementary material, further inquiries can be directed to the corresponding author.

## Ethics statement

The study was approved by the ethics committee of Shandong Provincial Hospital Affiliated to Shandong First Medical University (Approval No: SWYX: NO.2022-011). The studies were conducted in accordance with the local legislation and institutional requirements. The participants provided their written informed consent to participate in this study.

## Author contributions

ML: Writing – original draft, Methodology, Formal analysis, Data curation, Conceptualization. FQ: Project administration, Writing – original draft, Formal analysis. DW: Writing – review & editing, Methodology, Funding acquisition, Conceptualization.
